# Knowledge mapping of exosomes in ischemic stroke: a bibliometric analysis

**DOI:** 10.3389/fneur.2025.1595379

**Published:** 2025-07-23

**Authors:** Xiaofang Ding, Guoying Zou, Nuoya Ma, Xudong Tang, Jia Zhou

**Affiliations:** Department of Clinical Laboratory, The Second People’s Hospital of Hunan Province (Brain Hospital of Hunan Province), Changsha, China

**Keywords:** bibliometrics, exosomes, ischemic stroke, CiteSpace, VOSviewer

## Abstract

**Background:**

Ischemic stroke is a disease in which local ischemia and hypoxia of brain tissues are caused by obstruction of blood vessels in the brain, which in turn triggers brain tissue damage and neurological dysfunction. Recent studies have made significant progress in understanding the role of exosomes in ischemic stroke. Exosomes exhibit anti-inflammatory, immunomodulatory, anti-apoptotic, angiogenic, and neuroregenerative effects, as well as glial scar reduction and drug delivery effects in ischemic stroke. However, there is a notable gap in bibliometric analyses that focus specifically on this subject. This study systematically evaluated the current knowledge and identified emerging research trends regarding exosomes in ischemic stroke through a bibliometric analysis.

**Methods:**

We retrieved research articles on the role of exosomes in ischemic stroke published between 2004 and 2023 from the Web of Science Core Collection (WoSCC) database and then conducted a bibliometric analysis using VOSviewer, CiteSpace, and the bibliometrix package in the R programming environment.

**Results:**

A comprehensive analysis of 374 publications from 38 countries revealed a steady increase in research focused on exosomes in ischemic stroke. This analysis significantly emphasized the contributions of researchers from China and the United States. Key research institutions in this field include Henry Ford Health System, Henry Ford Hospital, and Shanghai Jiao Tong University. The *International Journal of Molecular Sciences* is the top journal in terms of publication output, and Stroke is the most frequently co-cited journal. This extensive study involved 468 authors, the most prolific of whom are Michael Chopp, Zhengbiao Zhang, and Liang Zhao, Hongqi Xin is the most frequently co-cited researcher. The primary areas of investigation are the role of endogenous exosomes in initiating and progressing ischemic stroke, as well as the potential therapeutic applications of exogenous exosomes.

**Conclusion:**

In the context of ischemic stroke, a recent bibliometric evaluation provided a comprehensive analysis of research trends and developments related to exosomes. The findings of this study highlight current research frontiers and identify significant emerging trends. These findings offer a crucial resource for researchers focusing on exploring exosomes.

## Introduction

Ischemic stroke significantly contributes to global mortality and disability (1). Treatment options are limited, primarily because effective interventions must occur within a short timeframe. This often results in suboptimal post-treatment outcomes. Therefore, it is crucial to investigate management strategies requiring immediate, comprehensive care. Current treatment options for ischemic stroke include thrombolytic therapy, mechanical thrombectomy, angioplasty, anticoagulant and antiplatelet medication use. In addition, various interventional techniques, such as stent placement and surgical revascularization, have been employed in clinical practice (2). However, it’s important to acknowledge that these surgical and interventional methods carry risks, and long-term medication use can result in side effects. Therefore, there is an urgent need to develop safer and more effective alternative treatments. It is particularly crucial to explore new strategies for managing ischemic stroke because timely intervention and a collaborative approach are essential for improving patient outcomes. Exosomes are small, 30- to 150-nanometer-sized vesicles released by various cell types into body fluids, such as blood, urine, saliva, and cerebrospinal fluid, they are formed by the fusion of endosomes with the plasma membrane, allowing the vesicles to enter the extracellular space. Exosomes carry a variety of important biomolecules, including lipids, proteins, RNA (especially microRNA and messenger RNA), and DNA fragments, all of which are essential for cellular communication and many biological processes (3). Almost all cell types can produce exosomes, which are characterized by low immunogenicity and tumorigenicity, efficient drug delivery, and blood–brain barrier crossing (4). Recent studies have shown that exosome therapy has neuroprotective and reparative effects in ischemic stroke, suggesting that it may be an effective new therapeutic strategy. Meanwhile, studies on using exosomes as diagnostic markers and using engineered exosomes as drug carriers are emerging. However, there is a gap between animal experiments and clinical translation, as well as between laboratory results and bedside applications. This paper uses bibliometrics to grasp the overall development of exosomes in ischemic stroke, reveal research hotspots, predict future research trends, accurately locate innovation breakthroughs, and provide “discipline navigation” and “cutting-edge insight” for the field, this information provides smarter decision support for the research ecology.

## Methods

### Search strategy

**Figure 1:**
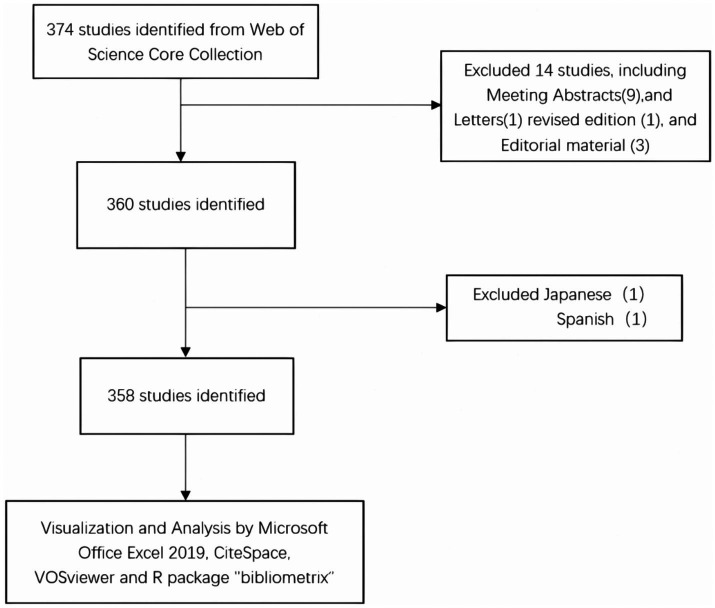
Publications screening flowchart.

A systematic literature search was performed in the Web of Science Core Collection (WoSCC),^1^ using the following query: ((TS = “Exosomes”) AND TS = “ischemic stroke”) AND LA = “English,” with filters applied for “articles” and “reviews” (Figure 1).

### Data analysis

VOSviewer (version 1.6.19) is bibliometric analysis software that extracts key information from numerous publications. It is commonly used to construct networks of collaboration, co-citation, and co-occurrence. In this study, the software performed the following analyses: journal and co-cited journal analysis, co-cited author analysis, country and institution analysis, and keyword co-occurrence analysis. In VOSviewer maps, nodes represent items such as countries, institutions, journals, and authors. The size and color of the nodes indicate the quantity and classification of these items, respectively. The thickness of the lines between nodes reflects the degree of collaboration or co-citation between items. In our study, CiteSpace was used to plot biplot overlays of journals and to analyze references using citation bursts. The R package “Bibliometrix” (version 4.3.2) was used for thematic evolution analysis and to construct a global distribution network of exosomes in ischemic stroke. Additionally, we analyzed the annual publication volume of papers using Microsoft Office Excel 2021.

## Results

### Quantitative analysis of publication

Our investigation identified 358 studies related to exosomes in the context of ischemic stroke published over the last two decades. This collection comprises 271 original research articles and 87 review papers. The data indicate that from 2012 to 2017, the number of publications was relatively low, averaging just 5.1 articles per year, which suggests that this research area was still in its early stages of development. In contrast, from 2018 to 2023, there was a remarkable increase in the publication frequency, with an average of 54 articles published annually. Notably, in 2020, 53 articles were published, 1.8-fold increase compared to previous years. The total number of publications reached 92 by 2023, demonstrating a consistent upward trend in research output since 2018, especially compared with earlier years.

### Country and institutional analysis

**Figure 2:**
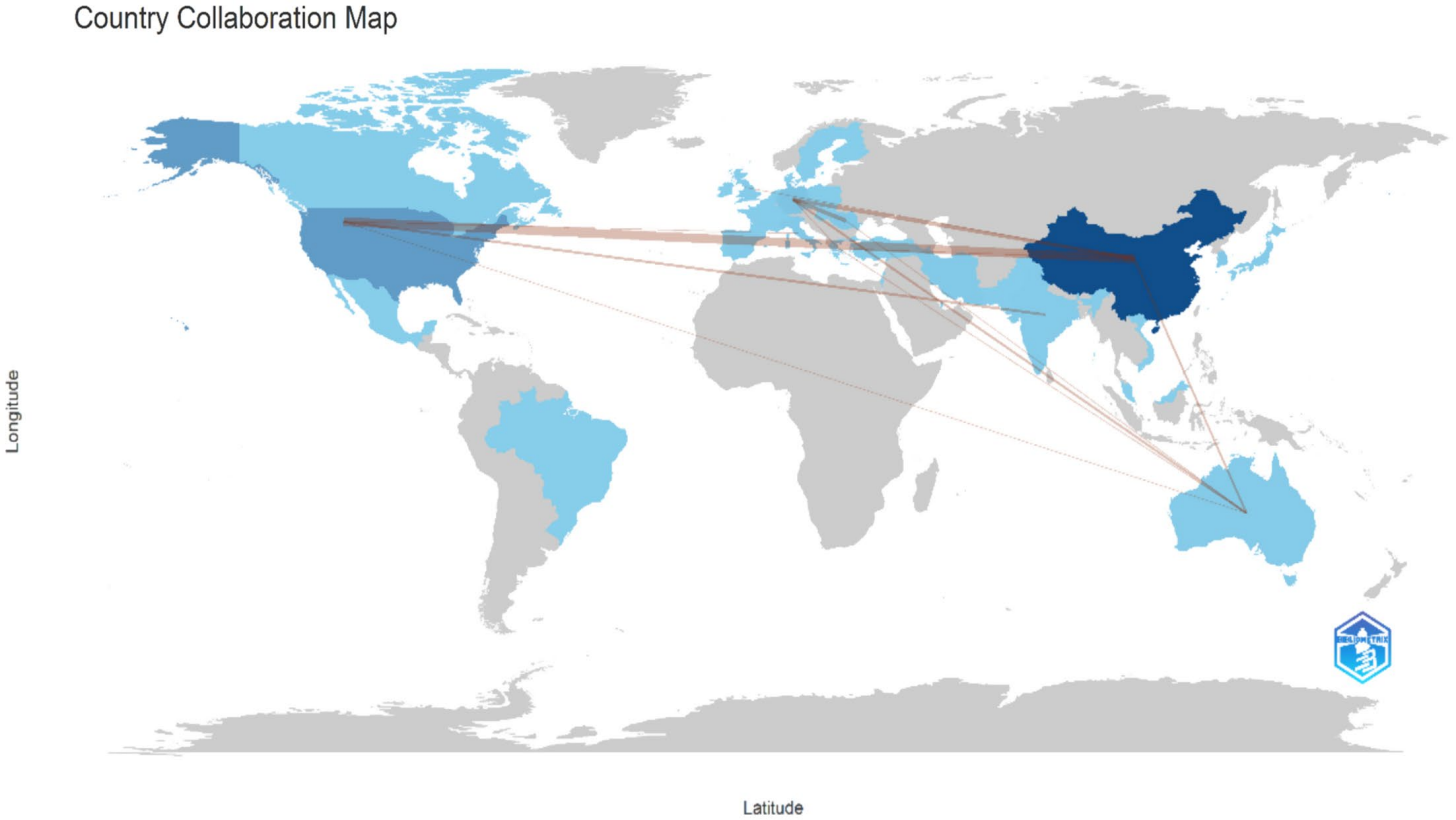
Geographical distribution of exosome research on ischemic stroke.

A total of 36 countries and 199 academic institutions have conducted research on the role of exosomes in ischemic stroke. A significant portion of this research originates from Asia, with notable contributions from three European countries (Table 1). China was the leading contributor, producing 229 publications, accounting for 59.48% of the total output. The United States was the second-largest contributor, with 85 publications (22.07%), Germany and Italy added 18 (4.6%) and 9 (2.3%) publications, respectively. China and the United States together constitute 81.6% of the total publication volume, an analysis of collaborative networks among 48 countries reveals strong partnerships, particularly between the two countries. Additionally, notable collaborations involve Germany and China, as well as Australia partnering with both (Figure 2).

**Table 1:** Top 10 countries and institutions on research on exosomes in ischemic stroke.

Rank	Country (region)	Publication counts	Institution	Publication counts (%)
1	China (Asia)	229	Henry Ford Health System (US)	20 (13.07%)
2	USA (North America)	85	Henry Ford Hospital (US)	20 (13.07%)
3	Germany (Europe)	18	Shanghai Jiao Tong University (China)	20 (13.07%)
4	Italy (Europe)	9	Oakland University (US)	19 (12.41%)
5	Romania (Europe)	9	Central South University (China)	15 (9.80%)
6	South Korea (Asia)	9	University System of Ohio (US)	13 (8.49%)
7	Iran (Asia)	8	Fudan University (China)	12 (7.84%)
8	Australia (Oceania)	6	Jinzhou Medical University (China)	12 (7.84%)
9	India (Asia)	6	Capital Medical University (China)	11 (7.18%)
10	Japan (Asia)	6	Nanjing Medical University (China)	11 (7.18%)

**Figure 3:**
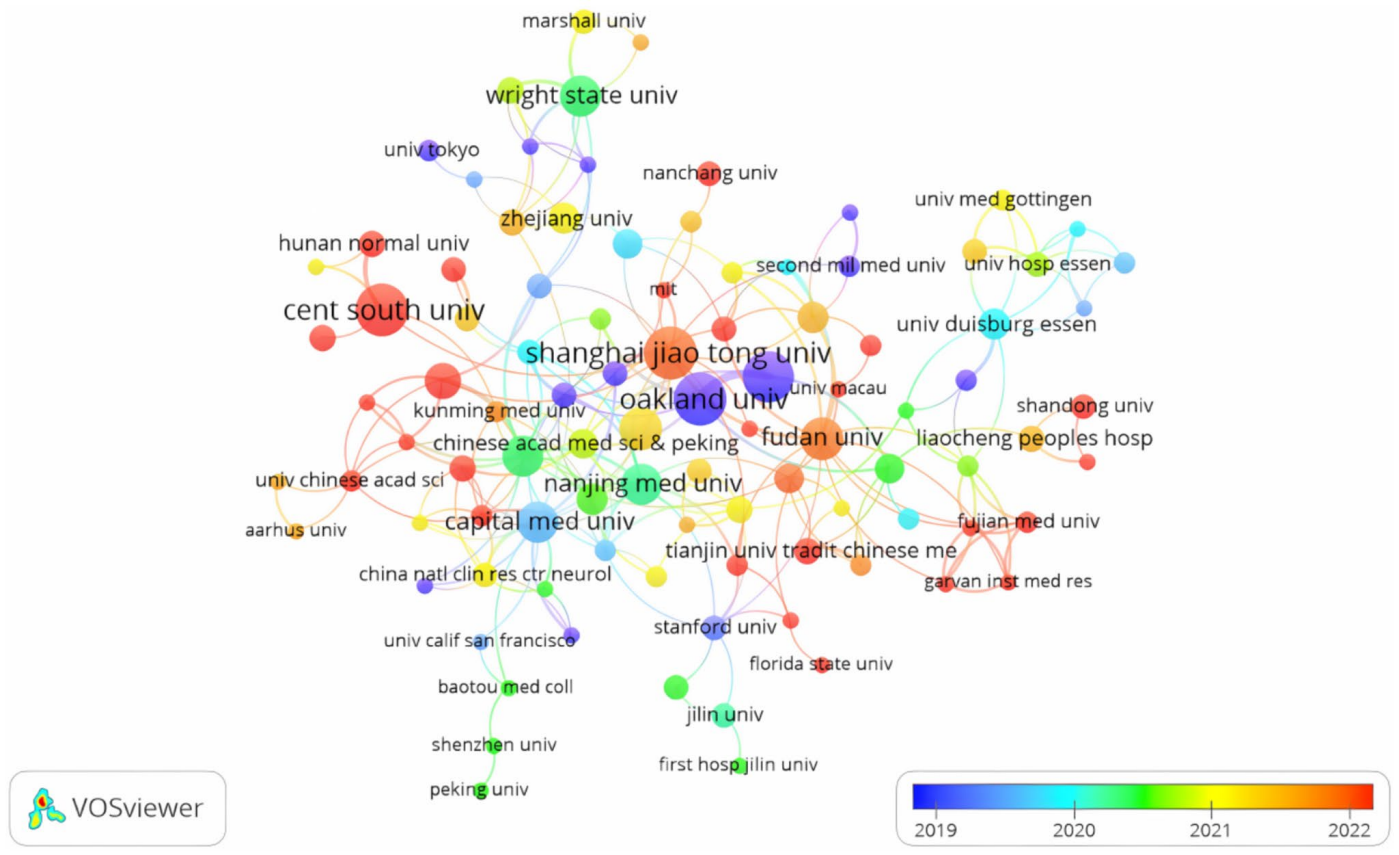
Visualization of institutions for research on exosomes in ischemic stroke.

The leading institutions in this field are primarily located in three countries, with China accounting for 60% of them. Notable contributors include the Henry Ford Health System, Henry Ford Hospital, Shanghai Jiao Tong University, and Oakland University. Each institution is responsible for about 13% of the total publications. This research focus resulted in the formation of a collaborative network of 62 institutions, each of which has published at least three papers, this highlights the significant cooperative efforts within this area. Key partnerships were formed between Shanghai Jiao Tong University, Fudan University, Tongji University, and Central South University. Additionally, Auckland University has established partnerships with Tianjin Medical University, Harvard Medical School, and Massachusetts General Hospital, highlighting the interconnected nature of research initiatives in this field (Figure 3).

### Journals and co-cited journals

**Figure 4:**
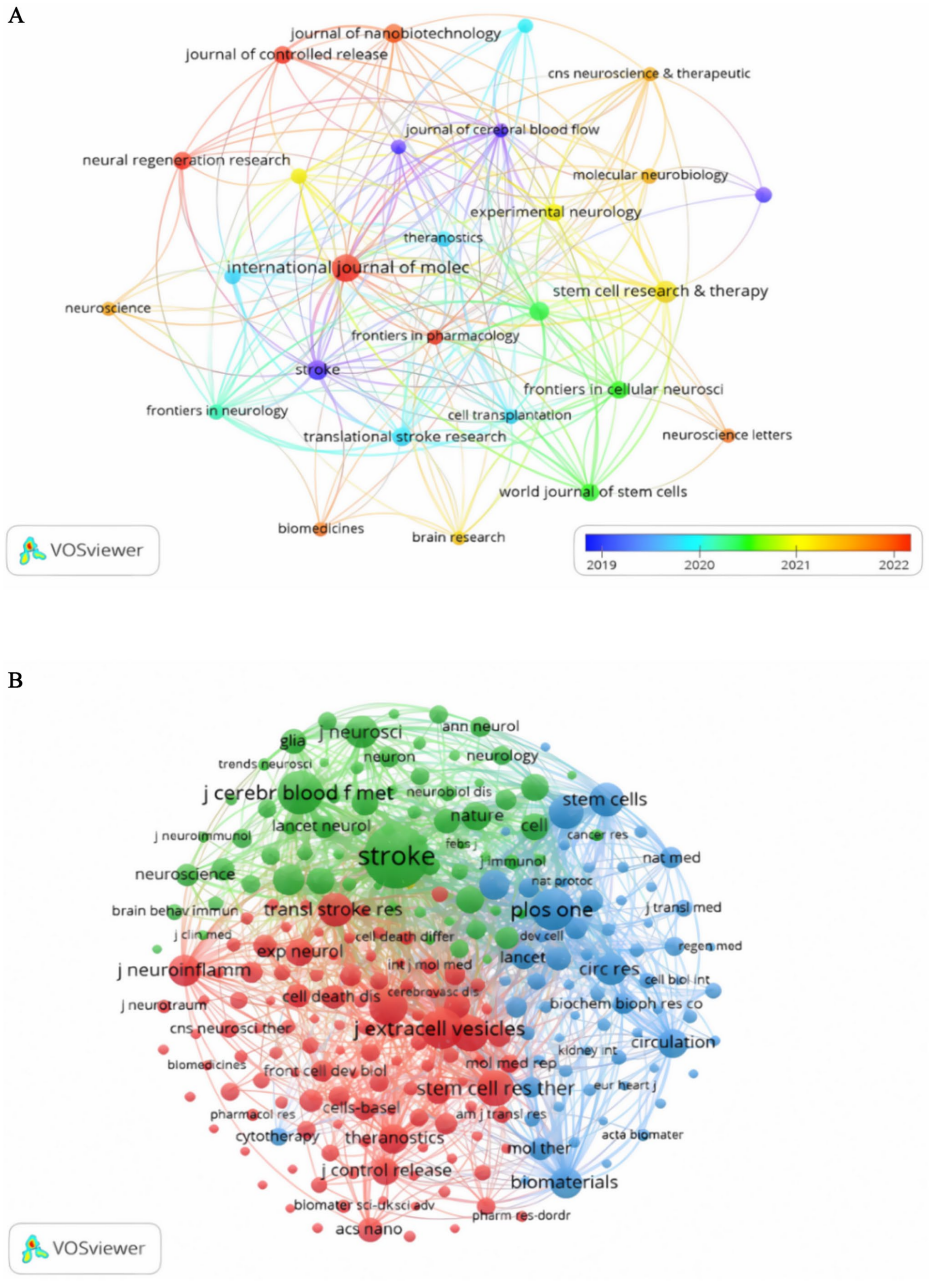
Visualization of journals **(A)** and co-cited journals **(B)** on research on exosomes in ischemic stroke.

**Figure 5:**
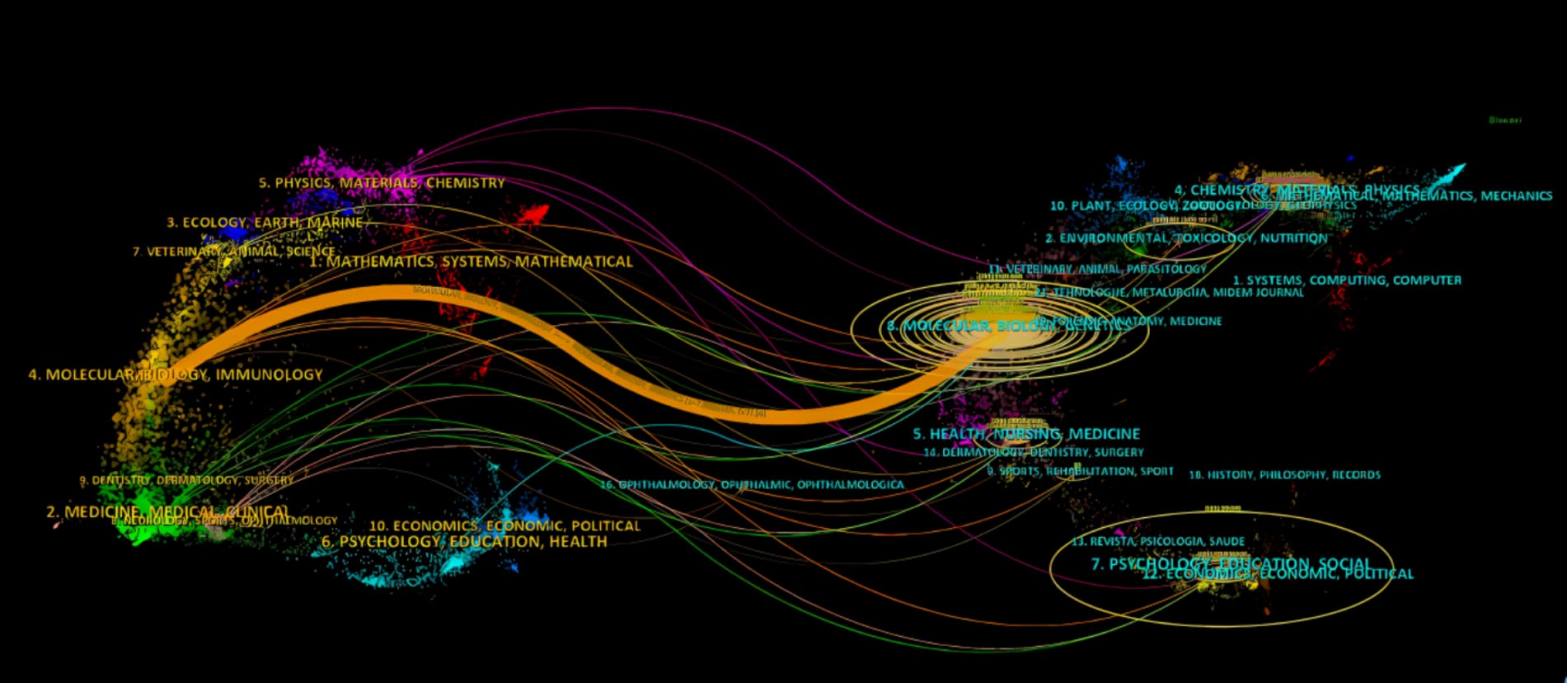
Dual-map overlay of journals on research on exosomes in ischemic stroke.

A thorough examination revealed that 178 scholarly journals published research on exosomes in the context of ischemic stroke. The *International Journal of Molecular Sciences* was the leading publication, accounting for 8.42% of the total output with 15 articles. *Stem Cell Research & Therapy* published 9 articles (5.05%), while *Stroke* and *Translational Stroke Research* contributed 8 (4.49%) and 7 articles (3.93%), respectively. Among the top 10 journals, the *Journal of Nanobiotechnology* had the highest impact factor at 10.6, closely followed by the *Journal of Controlled Release* at 10.5. A detailed literature review identified 27 journals, each with at least two relevant publications, which facilitated the creation of a citation network map (Figure 4A). This figure shows that leading international journals in the field of molecular science, such as *Stroke*, the *Journal of Cerebral Blood Flow and Metabolism*, and the *International Journal of Molecular Sciences*, demonstrate significant citation interactions within the established network. As shown in Table 2, four of the 10 most frequently cited journals surpassed 400 citations. Notably, *Stroke* leads the list with a co-citation count of 1,042, followed by the *Journal of Cerebral Blood Flow and Metabolism* (co-citations = 475), *PLoS One* (co-citations = 436), and the *Journal of Extracellular Vesicles* (co-citations = 408). In addition, *the Journal of Extracellular Vesicles* had the highest impact factor (IF = 15.5), followed by *the Proceedings of the National Academy of Sciences of the United States of America* (IF = 9.4). As shown in Figure 4B, *Stroke* exhibits positive co-citation associations with *the Journal of Cerebral Blood Flow and Metabolism*, *PLOS One*, *and the Journal of Extracellular Vesicles*, among others. This reflects the trend toward interdisciplinary cooperation and technological integration. The dual-map overlay of academic journals illustrates citation trends, with cited journals positioned on the left and co-cited journals on the right (5). The main citation path indicated by the orange line, reveals that research in the areas of *Molecular/Biology/Immunology* primarily references works from the *Molecular/Biology/Genetics* field (Figure 5).

**Table 2:** Top 10 journals and co-cited journals for research of exosomes in ischemic stroke.

Rank	Journal	Publication counts	Impact factor (IF)	JCR	Co-cited journal	Co-citation counts	Impact factor (IF)	JCR
1	International Journal of Molecular Sciences	15	4.9	Q2	Stroke	1,042	7.8	Q1
2	Stem Cell Research & Therapy	9	7.1	Q2	Journal of Cerebral Blood Flow and Metabolism	475	4.9	Q2
3	Stroke	8	7.8	Q1	PLoS One	436	2.9	Q3
4	Translational Stroke Research	7	3.8	Q2	Journal of Extracellular Vesicles	408	15.5	Q1
5	Frontiers in Neuroscience	7	3.2	Q3	International Journal of Molecular Sciences	346	4.9	Q2
6	Journal of Nanobiotechnology	7	10.6	Q1	Scientific Reports	335	3.8	Q3
7	Experimental Neurology	6	4.6	Q2	Stem Cell Research & Therapy	325	7.1	Q2
8	World Journal of Stem Cells	6	3.6	Q3	Stem Cells	299	4	Q2
9	Frontiers in Cellular Neuroscience	6	4.2	Q3	Translational Stroke Research	282	3.8	Q2
10	Journal of Controlled Release	6	10.5	Q1	Proceedings of the National Academy of Sciences of the United States of America	280	9.4	Q1

### Co-cited authors and co-cited references

**Figure 6:**
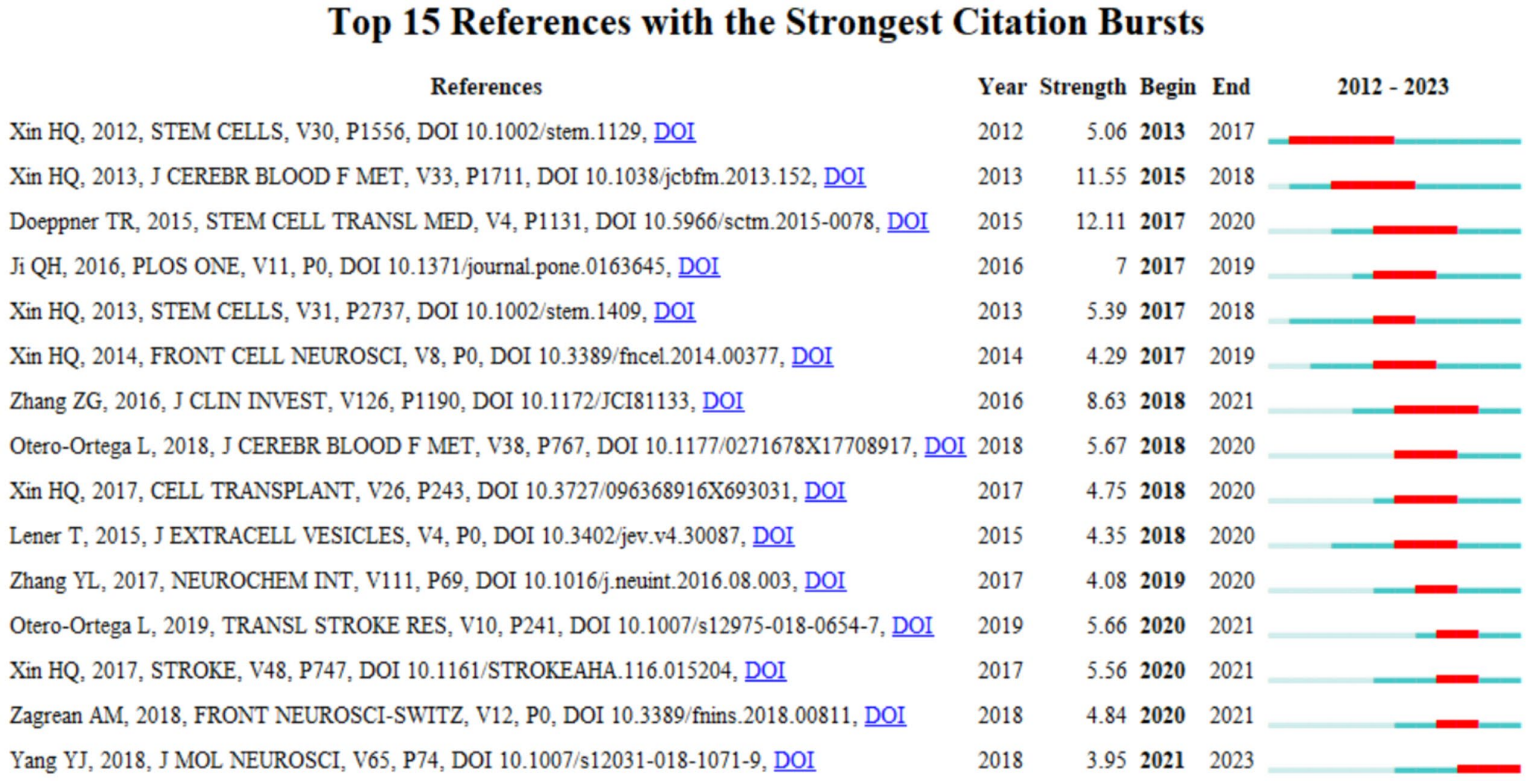
Top 15 references with strong citation bursts. The red bars indicate high citations in that year.

A total of 2,271 researchers have studied exosomes in the context of ischemic stroke. The top 10 authors among these contributors published 18, 10, 10, 10, and 10 articles, respectively (Table 3). Through the co-citation analysis, 13,032 authors were identified. Five of these authors had a co-citation frequency of more than 100 (Table 3). Leading this count was Xin H. Q., who received an impressive total of 367 citations, following Xin H. Q. was Zhang Z. G. with 129 citations, and Chen J. L. closely trailed with 124 citations. Furthermore, a co-citation network was established for authors cited at least 30 times (Figure 6B). This network highlights significant collaborative relationships, particularly between Xin H. Q. and Chen J. L., and between Xin H. Q. and Zhang Z. G.

**Table 3:** Top 10 authors and co-cited authors on research of exosomes in ischemic stroke.

Rank	Authors	Publication counts	Co-cited authors	Co-citation counts
1	Chopp, Michael	18	Xin, H. Q.	367
2	Zhang, Zheng Gang	10	Zhang, Z. G.	129
3	Hermann, Dirk M.	10	Chen, J. L.	124
4	Liang, Jia	10	Doeppner, T. R.	105
5	Zhao, Liang	10	Thery, C.	101
6	Yang, Guo-Yuan	9	Otero-Ortega, L.	88
7	Shi, Yijie	9	Zhang, Y.	80
8	Chen, Yanfang	8	Tian, T.	68
9	Zhang, Zhijun	8	Li, Y.	65
10	Xin, Hongqi	7	Yang, J. L.	63

Over the last 20 years, researchers have identified 17,271 co-cited studies pertaining to exosomes in the context of ischemic stroke. Within the top 10 most frequently co-cited references (Table 4), each publication received a minimum of 47 citations, with one reference surpassing 90 citations. These 10 articles demonstrate a changing research trend on exosomes: research on exosome is shifting from basic studies (existence, composition) to applications in disease treatment (e.g., stroke). This reflects basic research moving to clinical applications. Initially focused on cell biology, research on exosome involves neuroscience, material science, clinical medicine, and other disciplines, reflecting a transformation from a single field to multidisciplinary cross-fertilization.

**Table 4:** Top 10 co-cited references on research of exosomes in ischemic stroke.

Rank	Co-cited reference	Citations
1	Xin H. Q., 2013, J Cerebr Blood F Met, v33, p. 1711	93
2	Doeppner T. R., 2015, Stem Cell Transl Med, v4, p. 1131	73
3	Xin H. Q., 2017, Stroke, v48, p. 747	72
4	Valadi H., 2007, Nat Cell Biol, v9, p654	61
5	Xin H. Q., 2013, Stem Cells, v31, p. 2737	58
6	Tian T., 2018, Biomaterials, v150, p. 137	52
7	Zhang Z. G., 2019, Nat Rev Neurol, v15, p. 193	49
8	Xin H. Q., 2012, Stem Cells, v30, p. 1556	48
9	Yang J. L., 2017, Mol Ther-Nucl Acids, v7, p. 278	47
10	Song Y. Y., 2019, Theranostics, v9, p. 2910	47

### Reference with citation bursts

This study used CiteSpace to identify 15 pivotal publications characterized by citation bursts, which are notable spikes in citations within a specific period of time (Figure 6). The red bars in the figure denote the periods of increased citation activity that transpired from 2012 to 2021. The paper by Doeppner T. R. et al., regarding extracellular vesicles and their role in stroke recovery exhibited the most pronounced citation burst from 2017–2020, with an intensity of 12.11, proposing standardized protocols or efficacy enhancement strategies for stem cell transplantation that will serve as technical benchmarks for subsequent studies. A study by Xin et al., conducted from 2015 to 2018, focusing on exosomes and neurovascular plasticity post-stroke, recorded the second highest burst intensity of 11.55. The five high-bursts of literature published by Xin H. Q.’s team between 2012–2017 (e.g., Stem Cells, 2012) all focused on the mechanisms of stem cell transplantation for the repair of cerebral ischemia, suggesting that this direction was an early research hotspot. After 2018, the studies were more inclined to clinical validation (e.g., Otero-Ortega L., 2018) and molecular mechanisms (e.g., Zhang T. L., 2017), reflecting the transition of the field from fundamentals to applications.

### Hotspots and frontiers

**Figure 7:**
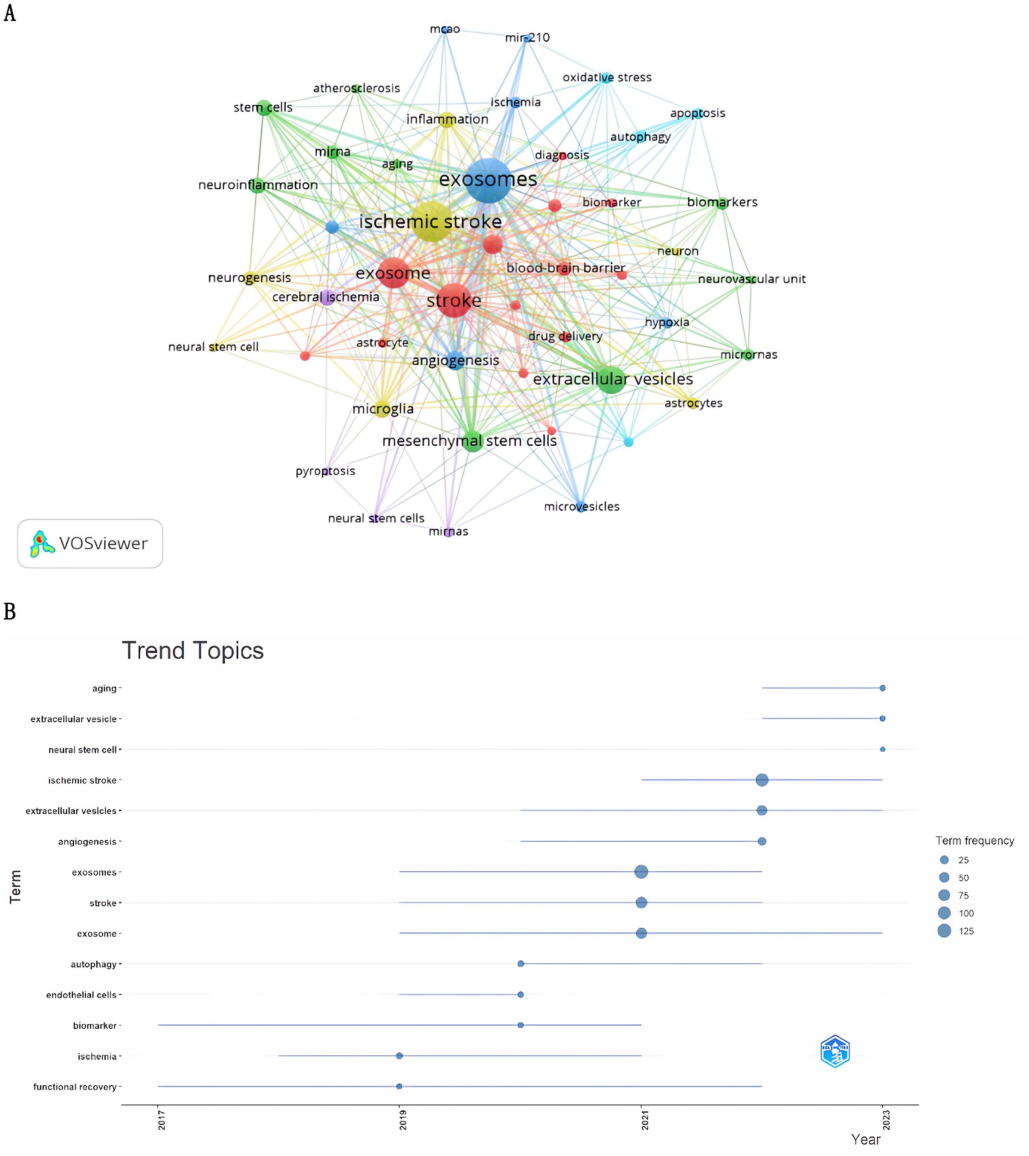
Keyword cluster analysis **(A)** and trend topic analysis **(B)**.

The analysis of co-occurring keywords highlights key research trends. Table 5 shows that “extracellular vesicles” and “mesenchymal stem cells” were the most cited keywords, each referenced more than 30 times, indicating their importance in ischemic stroke research. A study of keywords mentioned five or more times using VOSviewer (Figure 7A) revealed six clusters: the green cluster included extracellular vesicles, mesenchymal stem cells, microglia, angiogenesis, and stem cells; the red cluster covered exosomes, stroke, microRNA, and the blood–brain barrier; the blue cluster featured exosomes, angiogenesis, and neuroprotection; the yellow cluster consisted of ischemic stroke, microglia, inflammation, and neurogenesis; the purple cluster related to cerebral ischemia, miRNAs, and pyroptosis; and the cyan cluster involved autophagy, apoptosis, and oxidative stress. The trend analysis in Figure 7B shows that, from 2004 to 2019, research primarily focused on functional recovery and ischemic conditions. In contrast, after 2021, the focus has shifted to understanding the pathogenesis and therapeutic potential of exosomes, especially in aging, extracellular vesicles, and neural stem cells.

**Table 5:** Top 20 keywords on research of exosomes in ischemic stroke.

Rank	Keywords	Counts	Rank	Keywords	Counts
1	Exosomes	128	11	Inflammation	16
2	Ischemic stroke	104	12	Neuroinflammation	16
3	Stroke	74	13	Neurogenesis	13
4	Exosome	61	14	Blood–brain barrier	13
5	Extracellular vesicles	51	15	miRNA	12
6	Mesenchymal stem cells	31	16	Neuroprotection	11
7	MicroRNA	25	17	Biomarkers	11
8	Angiogenesis	25	18	Acute ischemic stroke	11
9	Microglia	20	19	Microvesicles	10
10	Stem cells	17	20	Oxidative stress	10

## Discussion

### General information

**Figure 8:**
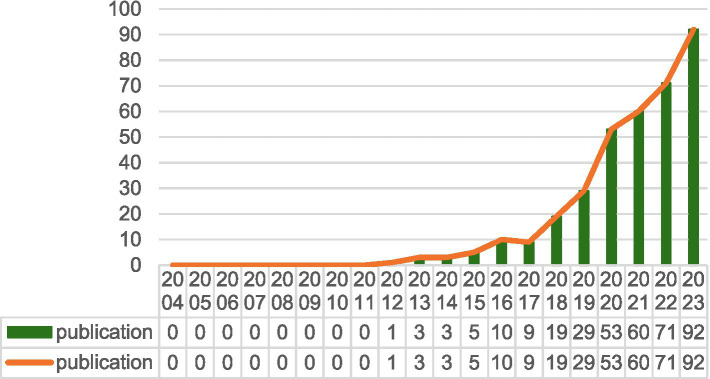
Annual output of research of exosomes in ischemic stroke.

Between 2004 and 2011, there were no publications concerning exosomes in the context of ischemic stroke (Figure 8). The immaturity of exosome isolation and purification technology, as well as the insufficient sensitivity of detection technology, makes it difficult to study the mechanism of action in depth. During the period when the biological function of exosomes was not well understood in academia, research hotspots did not focus on the application of exosomes in ischemic stroke. Insufficient multidisciplinary cross-fertilization and difficulties in clinical translation limited the research and application of exosomes in this field, indicating that there is an obvious gap in research linking these two fields during this period. From 2012 to 2017, the output of academic papers remained modest, with an average of about 5.1 publications per year, suggesting that exosome research is still in its infancy in this particular field. From 2012 to 2017, the number of academic papers published annually averaged about 5.1, suggesting that exosome research is still in its infancy. In contrast, from 2018 to 2023, there is a significant increase in published academic papers, averaging about 54 papers per year. A notable peak occurred in 2020, with 53 papers published, an 80% increase compared with 2019. Additionally, the number of published papers increased significantly to 92 in 2023. Over the past 6 years, the annual growth rate of research on exosomes and ischemic stroke has continued to increase, indicating a growing interest in this area that portends significant progress and increased academic attention.

**Figure 9:**
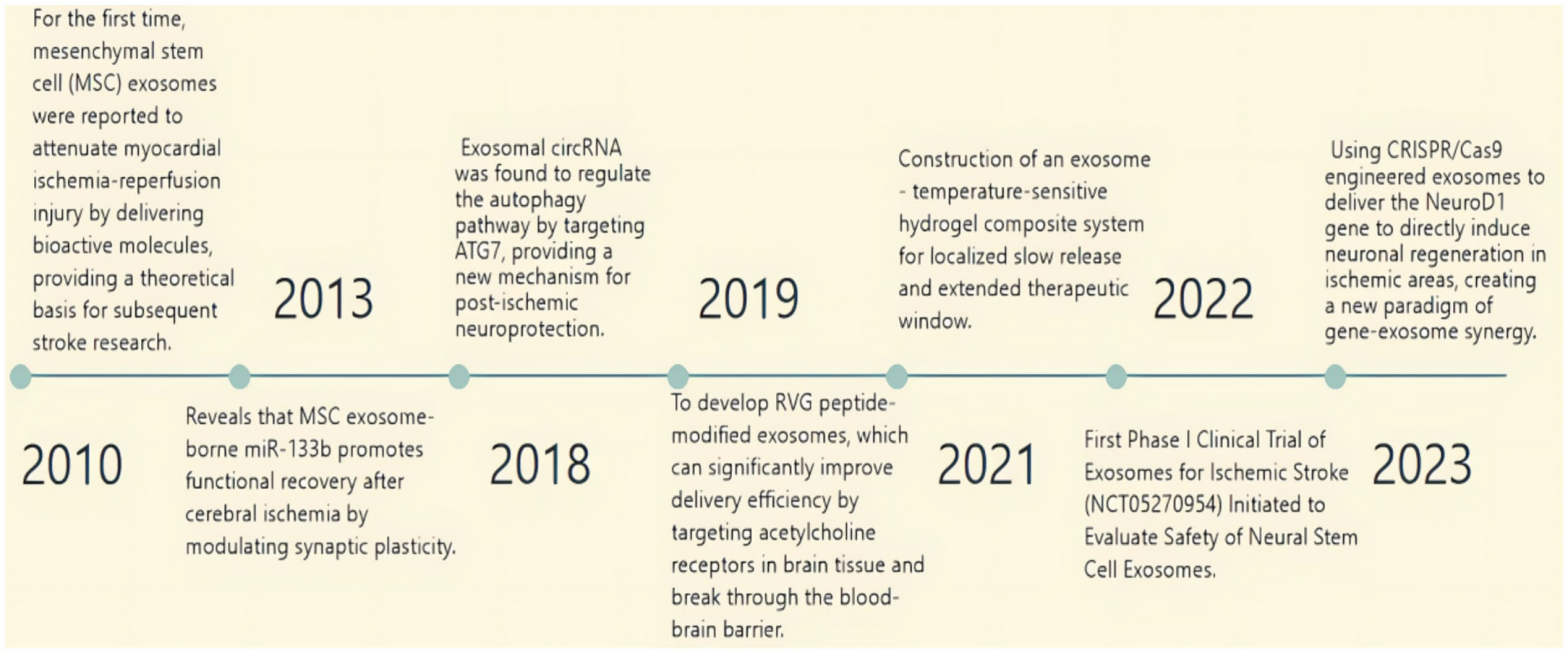
The evolution of exosomes in ischemic stroke applications.

The exploration of exosomes in the field of ischemic stroke, starting from the discovery of intercellular communication carriers and gradually revealing their multi-mechanism roles, has opened new avenues for the treatment of ischemic stroke, demonstrating the potential for clinical translation and moving towards clinical application. Since the discovery of the neurorestorative mechanism of exosomes in the early 2010s, research on exosomes in the treatment of ischemic stroke has gone through several phases of elucidation of molecular mechanisms, validation of animal models, and recent preliminary exploration in early-stage clinical trials, making exosomes a very promising option for therapeutic applications, especially in the treatment of ischemic stroke (Figure 9). As shown in Figure 8, the number of articles published each year about exosomes in ischemic stroke increased suddenly from 2019 to 2020. This increase is related to the outbreak of the novel coronavirus epidemic, which accelerated the application of exosomes in ischemic stroke therapy by promoting demand for mRNA delivery technology. This accelerated the research of inflammatory mechanisms and clinical translation, pushing exosome research from “basic exploration” to “clinical translation,” especially in the field of ischemic stroke, this has led to an upgrade in treatment concepts and technical tools. Technological breakthroughs and resource reorganization driven by crises can lead to interdisciplinary changes. To transform emergencies into sustainable drivers of innovation, there is a need for interdisciplinary collaboration, clinical orientation, and the development of a resilient research ecology. China and the United States are leaders in exosome research focused on ischemic stroke, with China ranking among the top countries in this field. Among the top 10 research organizations, 52.91% are in China, followed by the United States (34.63%) and New Zealand (12.41%). China and the United States dominate exosome research in ischemic stroke due to their significant advantages in basic research, technological innovation, clinical application, interdisciplinary cooperation, resource integration, policy support, and financial investment. Notably, China has significant collaborations with both the United States and Germany. Australia has also collaborated with both Germany and China.

Shanghai Jiao Tong University, Fudan University, Tongji University, and Central South University are among the research institutions that have formed strong partnerships. Institutions such as Auckland University, Tianjin Medical University, Harvard Medical School, and Massachusetts General Hospital have maintained active international partnerships. Despite the sizable number of papers from Central South University, its collaborative network is limited, highlighting the need for greater international cooperation. Therefore, to accelerate exosome research related to ischemic stroke, research institutions around the world should strengthen their collaboration.

A significant portion of the literature concerning exosomes in the context of ischemic stroke has been published in the *International Journal of Molecular Sciences* (IF = 4.9, Q1), highlighting their significance within this research domain. Among the journals with the highest impact factors, the *Journal of Nanobiotechnology* (IF = 10.6, Q1) holds the leading position, closely followed by the *Journal of Controlled Release* (IF = 10.5, Q1). Co-citation analysis revealed that the most cited journals were high-impact Q1 publications, underscoring the quality and global recognition of research in this field. The majority of published studies are found in journals dedicated to molecular biology and related fields, whereas clinical research journals feature relatively few articles. This suggests that the investigation of exosomes in the context of ischemic stroke is predominantly at the fundamental research level.

Chopp, Michael is the most published authors with 18 articles, closely followed by Zhang, Zheng Gang, Hermann, Dirk M and Liang, Jia, who each published 10 articles. Notably, 9 of the 18 articles authored by Chopp, Michael were devoted to the therapeutic significance of microRNAs (miRNAs) and various bioactive compounds found in exosomes in promoting stroke recovery. Their research showed that miR-27a, which is found in exosomes or small extracellular vesicles (sEVs) from cerebral endothelial cells (CEC-sEVs) in ischemic brain tissue, plays a crucial role in promoting axon growth and aiding brain remodeling. Additionally, a collaborative review by Zhang et al. highlighted the essential role of miRNAs in brain repair via exosome-mediated cellular communication. The review suggests that exosomes play a vital role in brain remodeling and offer promising opportunities for treating ischemic brain injury and improving neurological function. Dirk M. Hermann’s article titled “New Light on the Horizon” in Stroke describes how extracellular vesicles (EVs) in the blood can be a diagnostic tool for transient ischemic attack (TIA) and stroke. Liang and Jia highlighted the neuroprotective effects and functional improvements associated with exosomes, demonstrating their potential as a therapeutic option for ischemic stroke through specific antioxidant pathways.

In summary, the studies mentioned above have mainly discussed the pathogenesis, diagnosis, and therapeutic role of exosomes in ischemic stroke.

### MSC-exos

Mesenchymal stem cells (MSCs) have recently received significant attention due to their impressive tissue regeneration and immunomodulation capabilities, especially in ischemic stroke treatment. This increased interest stems from the ability of MSC-derived exosomes to cross the blood–brain barrier, their low immunogenic profile, and their minimal toxicity (6). Consequently, MSCs are being increasingly integrated into clinical trials focused on managing ischemic stroke, with numerous studies reporting favorable therapeutic outcomes. Consistent evidence shows that MSC-based treatments substantially improve recovery following ischemic stroke (7–9). MSCs can easily be isolated from various sources, such as the umbilical cord, bone marrow, and peripheral blood (10). These stem cell-derived exosomes exhibit characteristics such as low immunogenicity, reduced risk of tumor formation, high transport efficiency, inherent stability, and the capacity to traverse the blood–brain barrier (11, 12). Exosome-based therapies are still in the early stages of clinical application, but they have shown promising therapeutic effects in animal models of ischemic cerebrovascular accidents (CVAs) (13). Research indicates that exosomes from bone marrow mesenchymal stem cells (BMMSCs) can significantly alleviate systemic immune suppression 4 weeks after ischemia, they can also promote neurovascular regeneration and enhance motor function (14). Additionally, studies have shown that exosomes extracted from adipose-derived mesenchymal stem cells (ADMSCs) can reduce infarct size, promote neurological recovery, enhance corticospinal tract integrity, and promote white matter repair in rat stroke models (15). Furthermore, it has been shown that exosomes from BMMSCs are effective in reversing peripheral immunosuppression after ischemia, thereby promoting infarct neurovascular regeneration (16, 17). Exosomes derived from neural stem cells reduce volume and enhance recovery following stroke (18). In addition to MSC-derived exosomes, exosomes from other cell types have also been found to contribute to neuroprotection after stroke (19). Webb et al. (18) discovered that astrocyte-derived exosomes inhibited the expression of TNF-α, IL-6, and IL-1β, which attenuated neuronal damage by inhibiting autophagy.

### Exosomes and biomarkers

Exosomes are vesicles derived from body fluids, such as serum, plasma, and urine. Among their components, miRNAs are among the most widely studied (20). Exosomes contain various functional RNA molecules, including miRNAs, which reflect the physiological and pathological characteristics of the originating cells (21, 22). By transferring mature miRNAs to recipient cells, exosomes can modulate gene expression and affect various cellular and molecular pathways (23). Recent studies have highlighted the role of exosomal miRNAs in modulating physiological and pathological processes after ischemic stroke, as well as their contribution to brain remodeling by enhancing substance transport (24). Therefore, exosomes are considered promising biomarkers for early diagnosis and prognosis of stroke, as well as potential drug candidates for stroke therapy (25, 26).

Exosomal miRNAs are more stable than free miRNAs because they are shielded from enzymes and RNases in biological fluids, making them less likely to break down (27). The increased stability of exosomal miRNAs has made it possible to identify changes in their expression over time during disease progression. Additionally, it allows these microRNAs to promote sustained cellular signaling associated with disease (28, 29). Some researchers have investigated whether the transfer of miRNAs via exosomes creates a new mechanism of cell-to-cell communication (30). Exosomal miRNAs from the central nervous system may carry information from their parent cells. In contrast, exosomal miRNAs from injured neuroblasts can monitor the condition of brain cells and tissues directly (31–33). Recent experiments have revealed substantial changes in the synthesis, secretion, and composition of exosomes, suggesting potential new targets for disease treatment (34, 35). Exosomes are rich in miRNAs, which are more accessible than cellular miRNAs. Deep sequencing results indicate that the percentage of miRNAs present in serum exosomes is three to four times higher than that in pure serum (36). Consequently, exosomes obtained from biological fluids, along with their miRNA content, are increasingly recognized as important targets for biomarker analysis (37–39). Research suggests that in diseases of the central nervous system such as stroke, the sorting mechanisms of miRNAs during exosome biogenesis may be disrupted, which in turn affects both disease pathogenesis and neuroregeneration (40). Consequently, exosomal miRNAs may exhibit greater disease specificity than cellular or free miRNAs, they are considered superior biomarkers for stroke due to their sensitivity and specificity (41–43).

### Therapeutic and neuroprotective roles of exosomes

Exosomes can cross the blood–brain barrier (BBB) or choroid plexus, allowing information exchange between the central nervous system (CNS) and the peripheral circulation (44). The primary mechanism through which exosomes exert their therapeutic effects is molecular delivery, specifically via miRNA transfer (45). Exosomes facilitate communication between cells and tissues by delivering proteins and miRNAs (46). Exosomes regulate gene expression and various cellular and molecular pathways by releasing mature microRNAs (miRNAs) into recipient cells (47). The unique characteristics of miRNAs in exosomes allow them to serve as effective drug carriers, they can target the CNS specifically and modulate gene expression related to disease. This could guide the development of new therapeutic strategies for CNS disorders (48, 49). Exosomes can be administered via several routes, such as nasal, intravenous, intraperitoneal, and intracranial, to deliver proteins and RNA to the brain (50). This flexibility makes exosome-based drug delivery a promising method for treating central nervous system (CNS) diseases (51, 52).

Brain recovery after an ischemic stroke is achieved through various interconnected processes, such as the formation of new blood vessels and neurons, the production of cells that support myelin, the activation of mechanisms that prevent cell death, and the engagement of the immune response (53, 54). These processes work together to enhance the reconstruction of the neurovascular units and restore neurological function (55, 56). Research indicates that enhancing miR-126 significantly increases the therapeutic effectiveness of exosomes obtained from endothelial progenitor cells (EPCs) (57, 58). After an ischemic stroke, an insufficient blood supply can cause miR-126 to target vascular cell adhesion protein 1 (VCAM-1), thereby regulating EPC function and angiogenesis (59). Adipose-derived stem cell exosomes are rich in microRNA-181b-5p, which regulates angiogenesis after a stroke by inhibiting transient receptor potential melastatin 7 (TRPM7) (60). Neuronal exosomes can carry microRNA-132 (miRNA-132) to endothelial cells, which helps maintain the integrity of the blood–brain barrier (BBB) (61). Additionally, exosomes from human microvascular endothelial cells contain the Dll4 protein, which regulates angiogenesis. The Dll4-Notch signaling pathway, which occurs in both endothelial cells and pericytes, is essential for angiogenesis and for maintaining the integrity of the blood–brain barrier (62).

Neural regeneration and angiogenesis are key processes in recovery from ischemic stroke. Research on exosomal miRNAs is advancing rapidly, particularly in the field of nerve regeneration. Recent studies have demonstrated the ability of exosomal miRNAs to positively impact nerve injury by modulating apoptosis, the inflammatory response, and regenerative processes in nerve cells. These small RNA molecules are important cell-to-cell signaling molecules that regulate the growth, survival, and regeneration of neurons. Recent studies have shown that, in addition to serving as carriers for drug delivery, exosomes regulate signaling pathways related to nerve regeneration through the miRNAs they contain. For instance, researchers discovered that exosomes from adipose-derived stem cells promote Schwann cell proliferation and migration by delivering miRNA-22-3p, thereby accelerating the repair of peripheral nerve injuries (63). Additionally, another study demonstrated that Schwann cell-derived exosomes promote nerve regeneration and functional recovery via microRNA-21 (64). Studies have shown that exosomes containing miRNA-124 reduce ischemic injury by converting neural progenitor cells into neurons (65, 66). Mesenchymal stem cell (MSC) exosomes deliver microRNA-133b (miRNA-133b) to neurons and astrocytes, this leads to the downregulation of connective tissue growth factor (CTGF) and the secondary release of astrocyte exosomes, which promote synaptic growth (67). This action reduces PTEN levels and increases Akt and mTOR phosphorylation by activating TLR7 and NF-κB/MAPK pathways, ultimately promoting neurogenesis, neuroplasticity, and oligodendrocytopoiesis after ischemic brain injury (68, 69).

Inflammation is a key pathogenic mechanism of post-ischemic brain injury and a trigger of secondary injury. Exosomes derived from MSCs can ameliorate inflammation after acute ischemia or ischemia–reperfusion injury by modulating anti-inflammatory molecules (IL-4 and IL-10) and pro-inflammatory cytokines (IL-6, TNF-α, and IL-1β), and inhibiting microglia activation (70). Exosomes enriched with miR-138-5p or miR-1906 inhibit inflammatory signaling pathways and reduce inflammation, thereby enhancing recovery after stroke (71–73). Wang et al. (74) reported that Fxr2 in ADSC-derived exosomes alleviated iron-induced ferroptosis in M2 microglial cells by regulating the expression of Atf3/Slc7a11, which suppressed the inflammatory microenvironment and improved neurological recovery from brain I/R injury. Research shows that exosomes from bone marrow-derived mesenchymal stem cells (BMMSCs) can reduce ischemia–reperfusion injury by inhibiting inflammation and apoptosis mediated by the NLRP3 inflammasome (75, 76).

There are many challenges with using exosomes for the treatment and diagnosis of ischemic stroke. In terms of cell source, extraction, and purification, various cell types have different advantages and disadvantages. The extraction methods are time-consuming, costly, and complicated. Research on the mechanism of action is insufficient, and the synergistic effect is unknown (77). In clinical application, it is difficult to guarantee yield quality, and targeting is insufficient, affected by individual differences, safety and efficacy need to be verified (78, 79). In the future, it’s necessary to analyze the heterogeneity in conjunction with new technologies, conduct clinical trials to validate them and promote their use.

## Conclusion

Ischemic stroke is a serious neurological disease with an extremely complex pathogenesis, and its core pathology includes multisystem disorders such as inflammatory response, apoptosis, oxidative stress, and blood–brain barrier disruption. In recent years, exosomes have emerged in ischemic stroke research due to their unique intercellular communication function.

As nanoscale membrane-structured vesicles, exosomes are rich in active molecules such as proteins, lipids, and nucleic acids, and different sources of exosomes exhibit different modes of action in the stroke process. Neurogenic exosomes carry neurotrophic factors and precisely regulate inflammatory and apoptotic pathways to achieve neuronal repair and regeneration, while immunogenic exosomes play dual roles in early inflammatory amplification and late immune remodeling. In addition, the specific molecular markers carried by exosomes are expected to overcome the traditional diagnostic limitations due to their high specificity and sensitivity, opening a new window for early and accurate diagnosis and dynamic monitoring of ischemic stroke. However, there are still many bottlenecks in current research: the isolation and purification technology needs to be innovated to meet the needs of clinical translation; the metabolism and molecular regulation mechanism of exosomes in the brain needs to be thoroughly analyzed; and the stability, targeting, and safety of exosomes as therapeutic carriers need to be verified in large-scale clinical trials.

Although exosomes have the potential to serve as biomarkers and therapeutic vehicles in the diagnosis and treatment of ischemic stroke, their clinical translation still faces challenges. These challenges include standardization, targeted delivery, and large-scale production. In order to transition from laboratory research to clinical application, multidisciplinary innovation and rigorous, evidence-based validation are necessary.

## Data Availability

The raw data supporting the conclusions of this article will be made available by the authors, without undue reservation.

## Footnotes

^1^ https://webofscience_clarivate_cn.hnucm.opac.vip/wos/woscc/basic-search
